# The Ideal Doctor*Read at Meeting Central Texas Medical Society, Waco, January 10, 1900.

**Published:** 1900-02

**Authors:** N. A. Olive

**Affiliations:** Waco, Texas


For Texas Medical Journal.

### The Ideal Doctor.*

N. A. OLIVE, M. D., WACO, TEXAS.

It was the intention, at first, to give a brief presentation of Dr. Ideal, from every conceivable standpoint; but with further consideration we found that to view him from the standpoint of his personality; his literary as well as scientific attainment, to deal with him at the bedside; to follow him to his library and find him poring over one authority after another in pursuit of one additional scientific truth; so that he may discover one more fact to strengthen a hope for the prolongation of a useful life; to analyze the nobler impulses of his soul, to go with him to the hovel where he administers his charities almost as often as his medicine, to examine various authorities, familiarity with which is demanded of him, it could not be said to require the scope of a good-sized volume, but would be the work of a life time, and the earnest work that has characterized this unselfish man's daily conduct. While we will deal mainly with his personality, it is not to be forgotten that he has a good general knowlsary and no more. He will not incorporate the entire pharmacopœia cal processes of the organs of the body, and is able to recognize a pathological condition when encountered. He administers medicine, not for the fact that he was summoned, but to relieve abnor-

*Read at Meeting Central Texas Medical Society, Waco, January 10, 1900.

mal conditions. He understands his remedy, gives all that is necessary and no more. He will not incorporate the entire pharmacœpia in one vile mixture and give it for all ills to which poor misguided flesh is heir. He will not wander from nature's wisely chosen path, but regards it as the God of Health, and recognizes the fact that its laws too often violated, lead but to physical disaster and imperil, if not destroy man's life. Dr. Ideal will not advertise, his works do that for him. No strong medical man, of well rounded character, capable and honest, who would not practice deception for the dollar, was ever known to advertise.

Dr. Ideal may be well informed in every branch of his profession and ignorant of the religious persuasion of his best paying patron. He is wise enough to know that one may be the largest Methodist and smallest doctor in town; that he may be the most orthodox Baptist and flaunt the hand bills of quackery in your face; he may be the most consistent Campbellite and know nothing of the therapeutic indication of aqua pura; that he may believe in justification by faith and be an ideal medical man; or he may believe there is nothing in the whole affair *but* faith, and it has nothing to do with his competency as a physician; that he may believe in the theory of evolution or he can embrace the dust theory of the origin of man and his qualification as a doctor will remain unaffected; that he may be agnostic in theology and a good physician; or one who deals daily with human life may be agnostic in both.

If a Christian, Dr. Ideal will not go to the amen corner for his patients, and when he offers up an earnest supplication for some brother practitioner's patient, he will not call to inform the sick of the fact; he considers it a private transaction between himself and his Maker, and to let the patient know it would add no efficacy to the prayer.

This doctor always seeks a congenial atmosphere and there are his fastest friendships. He will not compromise an old friend in hope of gaining a new. He is ever faithful to one and never mistrusted by the other. He meets all obligations promptly, to do which, he charges what his services are reasonably worth and collects promptly as possible. He is a member of all medical societies he can join and attend; the participates in their councils; he recognizes it as consultation with the best men of the profession, and he is always anxious to meet them; he knows if one lives to himself he will soon become narrow minded, he grows impatient with his own deficiencies and dissatisfied with his calling; he loses interest in his patients and they have reason to lose interest in him.

Dr. I is not a natural specialist; he recognizes the fact that all things do not come to man at once and nothing except at the cost of continuous hard work. He is singular in this respect, for many of his fellows live long lives, enjoy comparative success from a financial standpoint and die holding contrary views.

This doctor is an ideal husband, he has no secrets, professional ones excepted, too good for his wife to know. He is an ideal father, his devotion to his children is unbounded, his home is a paradise, to which he goes for recreation's happiest hours and the realization of love's loftiest ambition, her fondest hope.

His manners are polished, his action conscientious, his life consistent, and truth he esteems above all else.

The ideal doctor never refers to his own achievements, except when necessary and then in the most modest manner. He shrinks from any form of notoriety, he knows it comes too often without merit; he is happy in the knowledge that he merits all praise spoken of him and appreciates the fact that the honorable can bear unjust criticism better than can those of unsavory reputation.

The doctor is cleanly of person, avoiding all superfluous clothing and hair; he recognizes an unkempt beard as a carrier of infection, under his finger nails is no hot bed for the propagation and dissemination of all kind of disease germs; with him, cleanliness is indeed next to godliness.

This man is always apparently cheerful; he knows the keen eye of the patient is strained to read every look of disappointment on his face.

His habits are ideal as far as he can control them. When worked down he will not buoy up by artificial means, for the corresponding depression is sure to follow and will be permanently felt sooner or later. He takes absolute rest from his labors when demanded, thereby lives longer and renders mankind better service. In a crisis he is hopeful to the last; but will not let anxiety betray him into false hope and favorable prognosis when there is no ground for either. The doctor has almost perfect control of his emotions and never did make any vulgar demonstration that could be construed into an unnatural feeling for his patient, in hope of continuing in favor with the family. He will not rush forth and embrace and kiss every child he sees, for with the ignorant and filthy he will be defiled and with the intelligent and cleanly he can not be misunderstood. He is a good judge of human nature, but will not boast of never having been fooled in man. He knows that God's handiwork is beyond his comprehension and that he may be mistaken. He employs language to express ideas and to him oaths are meaningless.

He strictly fulfills every engagement and from his counsel others borrow wisdom; he takes pleasure in imparting knowledge. His opinion is sometimes worth having and is given in an unassuming manner to the one with whom he consults; but to the family, very sparingly, except through the attending physician, or at his request. He was never known to tarry with a patient when the attendant was gone, thus endeavoring to ingratiate himself, by unfair means (into favor), with the family and friends. Any mediocre can, at times, gain advantage of his superiors in the consultation room; only a consummate scoundrel will. Dr. Ideal is not intolerant of the imperfections of others, for to him, his own are the greater burden. He has a standard of right and lives to it. He is ambitious; but his ambition is to render others more happy than he found them. If he finds a home burdened with dissension, her hope departing, the light of love faded and flickering, the green-eyed monster gnawing at her vitals, he will not sever the frail thread that holds it together; but seeks to reunite the broken strands of the golden cord and strengthen the weakened ones. He lives to meet the approval of his fellow man and is mourned of all when gone.

His presence is sunshine; from his countenance beams the light of beneficence. He seeks knowledge from the humblest cottage as well as the gilded palace, and in his research of nature his reward is often great, where others fail to find. He is impatient of flattery and will not tolerate the unmeaning plaudits by which the weak are often beguiled. In short, he is honest, manly, generous, independent, energetic and capable.

He was born of intense desires but has a mastery of self. His unquenchable thirst for knowledge is never satiated. In his bosom burns the light of undying love; but the object of his contempt occupies a very small place in his mind. He is seldom astonished at a new truth but often astonishes the world by springing one upon her. He is the soul of affability and his courtesies are shared alike by great and small.

Dr. Ideal has the clearest possible conception of the object of human existence and his is the keenest regard for the preservation of life; be that during the first weeks of utero-gestation, or the last year of a long, useful career. And he has all modern appliances for the relief of pain and prolongation of life. His code is higher than any builded of words. He will not seek to cover an unmanly act by the apparent misconstruction of a vague word or sentence; for he knows language is faulty and has many meanings; but mind is its master and can have but one.

With him the code of medical ethics is not obsolete. It is to his profession what the constitution is to his country; it is the foundation of all order and should be adhered to until replaced by a more clearly defined declaration of principles.

In our daily life Dr. Ideal is more often discussed than met; but all agree that he should be oftener met.

Doctor's duties are such as are calculated to strengthen rather than weaken the character of man.

Let us hope that when we are gone and our imperfections buried, the generation to come will furnish more ideal doctors than has the present.

The hope that all doctors may be ideal is only to be realized by the slow process of evolution that has brought the human family to the proud position that it now occupies.

